# Developing Cancer Quality of Care Indicators to Quantify the Impact of a Global Destabilization of the Care System (COLLAT-COVID)

**DOI:** 10.3390/cancers17101680

**Published:** 2025-05-16

**Authors:** Nathalie Piazzon, Julie Haesebaert, Philippe Michel, Anne Sophie Belmont, Vahan Kepenekian, Gery Lamblin, Charlotte Costentin, Julien Péron

**Affiliations:** 1 Service de Gynécologie Hôpital de la Croix-Rousse, Hospices Civils de Lyon, 69004 Lyon, France; nathalie.piazzon@chu-lyon.fr; 2 Service de Recherche et Epidémiologie Cliniques, Pôle de Santé Publique, Hospices Civils de Lyon, 69424 Lyon, France; julie.haesebaert01@chu-lyon.fr; 3 Service Promotion, Prévention, Santé Populationnelle, Direction Qualité Usagers et Santé Populationnelle, Hospices Civils de Lyon, 69424 Lyon, France; philippe.michel@chu-lyon.fr; 4 Plateforme Transversale de Recherche Clinique de l’Institut de Cancérologie, Hospices Civils de Lyon, 69495 Pierre-Bénite, France; anne-sophie.belmont@chu-lyon.fr; 5 Service de Chirurgie Digestive et Oncologique, Hôpital Lyon Sud, Hospices Civils de Lyon, 69495 Pierre-Bénite, France; vahan.kepenekian@chu-lyon.fr; 6 Service de Gynécologie Obstétrique, Hôpital Femme-Mère-Enfant, Hospices Civils de Lyon, 69677 Bron, France; gery.lamblin@chu-lyon.fr; 7 Service Hépato-Gastroentérologie et Oncologie Digestive, CHU Grenoble Alpes, 38000 Grenoble, France; ccostentin@chu-grenoble.fr; 8 Service d’Oncologie Médicale, Hôpital Lyon Sud, Hospices Civils de Lyon, 69495 Pierre-Bénite, France

**Keywords:** COVID-19, crisis-responsive quality indicators (QIs), cancer pathologies, health system disruptions

## Abstract

Background/Objectives: The COVID-19 pandemic led to significant disruptions in healthcare systems, particularly impacting cancer care through delays in diagnoses and treatments. Quality indicators (QIs) are essential tools for monitoring healthcare performance, yet existing QIs may not be suited for crises. This study aimed to develop a set of hospital-based QIs tailored to assess the impact of care reorganization during health crises across four cancer types: breast cancer, hepatocellular carcinoma, gynecological cancers (excluding ovarian cancer), and peritoneal carcinomatosis. Methods: A multidisciplinary steering committee (SC) conducted a five-stage process, including a literature review, indicator selection, content validation via the RAND/UCLA method, final validation by the SC, and a pilot feasibility study. QIs were assessed based on clinical relevance, reproducibility, sensitivity to change, and feasibility. Expert panels evaluated and validated the indicators in two rounds of voting. Results: Among 150 initially identified QIs, 49 were validated: 12 for breast cancer, 11 for hepatocellular carcinoma, 8 for gynecological cancers, and 18 for peritoneal carcinomatosis. Most (92%) were process indicators, covering diagnosis, treatment, and care delays. Two common indicators were identified across all four cancers: multidisciplinary team meeting discussions and psychological support consultations. Conclusions: This study demonstrates the feasibility of developing crisis-responsive QIs to monitor cancer care during health system disruptions. Future work will focus on their real-time implementation, validation in international settings, and integration into healthcare policies to enhance crisis preparedness.

## Simple Summary

The COVID-19 pandemic severely disrupted healthcare systems, particularly affecting cancer care due to delayed diagnoses and treatments. To assess the impact of such crises, this study developed a set of hospital-based quality indicators (QIs) for four cancer types: breast cancer, hepatocellular carcinoma, gynecological cancers (excluding ovarian cancer), and peritoneal carcinomatosis. A multidisciplinary team followed a structured process, including a literature review, expert panel validation using the RAND/UCLA method, and final selection by a steering committee. Among 150 initially identified indicators, 49 were validated, with most focusing on care processes such as diagnosis, treatment, and therapeutic delays. Two indicators were common to all four cancers: multidisciplinary team discussions and psychological support consultations. This study highlights the feasibility of developing QIs tailored to health crises. The next steps will involve real-time implementation, international validation, and integration into healthcare policies to enhance crisis preparedness and ensure continuous quality improvement in cancer care.

## 1. Introduction

The COVID-19 pandemic, which began in 2020, had a significant impact on healthcare systems worldwide, leading to widespread lockdowns, travel restrictions, and reorganizations among healthcare providers to limit virus transmission and address increased pressure on health services.

In France, a series of successive and graduated national directives mandated extensive rescheduling of medical procedures across all healthcare establishments. On 16 March 2020, the government ordered the “postponement of any non-urgent surgical or medical activity, while taking into account potential risks to patient outcomes” [1]. Furthermore, during the initial lockdown, organized screening programs for breast, colorectal, and cervical cancer were suspended [2,3].

The effects on cancer care were particularly severe due to the potential negative consequences of delayed diagnoses, the complexities of cancer treatment pathways, the frequent need for high-risk surgeries and intensive care, and the heightened vulnerability of cancer patients to COVID-19 [4,5]. In early 2020, the first lockdown in France led to a dramatic decline in cancer screenings, diagnoses, and treatments, with the notable exception of chemotherapy [6,7,8,9]. In contrast, subsequent public health measures, including a second lockdown in late 2020, did not significantly impact cancer care delivery [10].

During times of healthcare system strain, as evidenced by the COVID-19 pandemic, monitoring care quality becomes crucial for informing and supporting local and national organizational initiatives. Real-time quality of care indicators were used to adapt health policies and provide operational guidance for healthcare systems [11,12]. Quality indicators assess specific healthcare processes or outcomes, and their key attributes include reliability (absence of measurement bias), validity (accurately measuring what they are intended to assess), relevance, actionability (usefulness for policymaking, monitoring, or strategy development), and feasibility [13]. A widely recognized conceptual framework for health system performance measurement, developed by the OECD, helps member countries prioritize areas for improving care quality [13].

Indicators that monitor cancer patient outcomes, such as five-year survival rates, are routinely used in many countries. While improving survival remains the ultimate goal, data on intermediate outcomes, processes, and healthcare structures are essential for guiding health system policies. A wide range of cancer quality indicators exists, covering aspects such as diagnosis, treatment, prevention, follow up, palliative care, rehabilitation, and even research [14,15]. However, in many countries, these indicators are not routinely available and require significant effort to collect, limiting their usefulness in informing real-time health system policies during crises, like the COVID-19 pandemic [16]. In this context, the development of indicators specifically adapted to disruptions in care delivery during global health crises may represent a relevant approach. The indicators developed should be designed not only as quality indicators but also as true Key Performance Indicators (KPIs), capable of guiding strategic decision-making during times of crisis. Indeed, when specifically designed to be sensitive to unstable contexts, quality indicators can serve a dual purpose: monitoring clinical practices while acting as strategic management tools for decision-makers [17,18]. These indicators, therefore, aim not only to assess the quality of oncology care but also to measure the responsiveness, resilience, and adaptability of healthcare structures. This hybrid positioning reflects an integrated approach that combines clinical relevance with organizational utility.

The primary challenge in creating cancer-specific indicators lies in the heterogeneity of cancer as a disease, with each tumor type following a distinct care pathway. This article aims to describe the process of identifying and developing a set of hospital quality of care indicators for four cancer types to monitor the impact of care reorganization during health crises.

## 2. Materials and Methods

This study was conducted in four regional hospitals: Grenoble University Hospital, Léon Bérard Cancer Center, Médipôle Lyon-Villeurbanne, and Hospices Civils de Lyon, ensuring that the impact of the epidemic on the Rhône and Isère regions was adequately represented.

In 2020, a multidisciplinary steering committee (SC) was established to oversee the project. The committee included two clinicians, a project manager, two public health specialists, and a biostatistician. The SC decided to develop quality indicators (QIs) for four patient cohorts corresponding to distinct cancer sites, each expected to exhibit significant heterogeneity in the impact of the COVID-19 crisis on patient workflows: breast cancer, hepatocellular carcinoma, gynecological cancers (excluding ovarian cancer), and peritoneal carcinosis. Ovarian cancer was excluded from the gynecological cancer cohort due to its natural history, which is characterized by frequent dissemination into the peritoneal cavity. In this study, a dedicated cohort for peritoneal carcinomatosis, regardless of the primary tumor site, was established. Therefore, including ovarian cancer in the gynecological cancer group was considered redundant. Its analysis was integrated into the peritoneal cancer cohort to ensure nosological and analytical consistency.

For each cohort, a clinical referent was appointed based on their expertise. The SC, in collaboration with the clinical referents, was responsible for assembling both the bibliography panel and the expert panel.

This study followed five key stages for each cohort as follows:

1. Literature Review: A bibliography panel, composed of two to three clinical experts supported by public health specialists and methodologists from the SC, conducted a literature review using PubMed and Google Scholar to identify relevant studies and reviews on QIs. Additional searches were conducted on official state websites and documents to gather indicators developed by national organizations recognized for promoting patient care and safety. For each identified QI, the panel recorded its title, calculation method, inclusion and exclusion criteria, and bibliographic references.

2. Selection of Indicators: Based on the indicators identified in the previous stage, the SC selected those deemed appropriate for this study. Indicators were excluded if they did not assess hospital-based quality of care, were too similar to others, or posed significant measurement challenges (e.g., involving multiple components or lacking clarity).

3. Content Validation: Content validation was conducted using the RAND/UCLA method [19,20], a modified Delphi technique involving a multidisciplinary panel of experts and anonymous scoring cycles. This method helps identify areas of agreement and disagreement among medical experts [20]. The validation process involved consensus opinions from an expert panel of 5 to 11 clinical specialists, along with a methodological expert. This study was conducted regionally, with participation from professionals across the four hospitals mentioned.

Two rounds of consensus were implemented. The first round was conducted via an electronic questionnaire sent by email. The second round took place through videoconferencing or in-person meetings. The questionnaire, created using the Mesydel platform (2021), included five closed questions per indicator, with responses measured on a 10-point Likert scale. The selection criteria assessed were (a) clinical relevance, (b) inter-institutional variability, (c) reproducibility, (d) sensitivity to change, (e) measurability within a short timeframe, and (f) suitability for assessing the impact of the COVID-19 crisis. Each indicator aimed to measure the effect of the COVID-19 crisis on care quality within the cohort.

Experts rated their agreement with the six selection criteria on a 9-point Likert scale (1 = strongly disagree, 9 = strongly agree). A criterion was validated if it achieved a median score of ≥7 and demonstrated consensus among voting members (“consensus to retain”). An indicator was considered validated when all selection criteria met the required threshold.

QIs that failed to achieve validation in the first round were reviewed in the second round. Experts were provided with a summary of their initial responses, along with anonymous group responses, to facilitate informed re-evaluation. After discussing criteria that had not reached consensus, experts were invited to re-vote on the same selection criteria.

4. Final Selection: QIs that achieved validation for clinical relevance and sensitivity to change but did not meet other criteria were submitted to the SC for a final decision. The final set of selected QIs was shared with both the bibliography panel and the expert panel for feedback.

5. Pilot Study: A pilot study for reliability analysis is currently ongoing and falls beyond the scope of this manuscript.

## 3. Results

### 3.1. Quality Indicator Selection Process

The selection of quality indicators (QIs) took place between November 2020 and June 2021. A total of 11 clinicians participated in the bibliographic panels, with representation as follows: breast cancer (*n* = 3), hepatocellular carcinoma (*n* = 3), gynecological cancer (*n* = 3), and peritoneal carcinomatosis (*n* = 2). Each panel was supported by at least one methodologist (Table 1).

**Figure 1:**
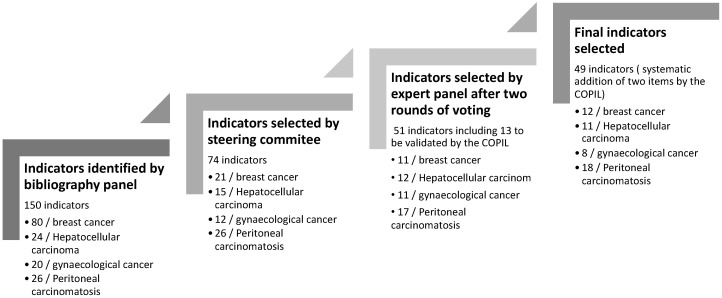
Indicator selection process.

The bibliographic panels initially identified 150 indicators: 80 for breast cancer, 24 for hepatocellular carcinoma, 20 for gynecological cancer, and 26 for peritoneal carcinomatosis. Following discussions, the steering committee selected 74 potential indicators and submitted them to expert groups for content validation. Although the bibliographic panels worked independently, several QIs were common across all four cancer types, particularly those related to care relevance, delays between diagnosis and therapeutic procedures, treatment modalities, and management processes (Figure 1).

A total of 23 clinical experts participated in the expert panels, distributed as follows: breast cancer (*n* = 6), hepatocellular carcinoma (*n* = 7), gynecological cancer (*n* = 6), and peritoneal carcinomatosis (*n* = 5). The expert panels validated 38 indicators after two rounds of voting. Additionally, 13 more QIs were considered valid based on clinical relevance and sensitivity to change, even though they did not meet all selection criteria. Ultimately, the steering committee selected 49 indicators (Figure 1).

### 3.2. Nature of Validated Indicators

The validated indicators covered all phases of the patient care pathway and were categorized according to the three domains of the Lancet Global Health High-Quality Health Systems framework: foundation, care process, and quality impact [21]. The foundation domain includes the facilities, personnel, and tools necessary for delivering care. The care process domain encompasses indicators related to competent, timely, and effective care, as well as patient experience. The quality impact domain reflects positive health outcomes, such as reductions in morbidity and mortality.

A significant majority of the selected QIs (92%) focused on monitoring hospital quality of care through the care process, while only 8% pertained to quality impact. Notably, no foundation indicators were validated in this study.

**Figure 2:**
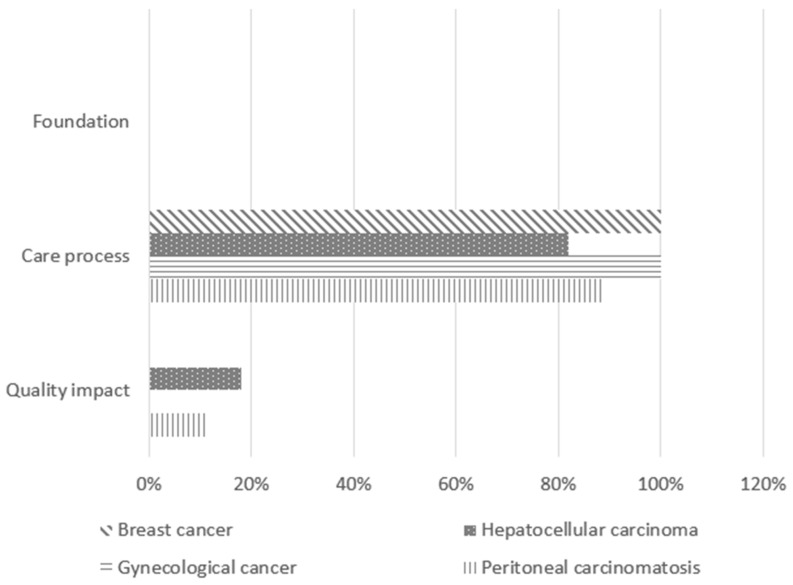
Nature of the validated quality indicators.

Despite variability in indicators by disease type, there were notable similarities in the proportions of different indicator types selected. Specifically, indicators related to the care process were particularly well represented, with proportions ranging from 82% to 100% (Figure 2).

The twelve QIs selected for the breast cancer pathway (see Appendix A, Table A1) were all process indicators: three focused on the diagnostic process, three on treatment modalities, five on delays before or between treatments, and one on staging.

The hepatocellular carcinoma pathway included eleven indicators (see Appendix A, Table A2), comprising two quality impact indicators and nine care process indicators: three focused on the diagnostic process, five on treatments, and one on delays before or between treatments.

The gynecological cancer pathway (excluding ovarian cancer) featured eight process indicators (see Appendix A, Table A3): three related to the diagnostic process, three to treatments, and two to delays before or between treatments.

The peritoneal carcinomatosis pathway included eighteen indicators (see Appendix A, Table A4), of which two were quality impact indicators and sixteen were process indicators: one focused on the diagnostic process, nine on treatments, and six on delays before or between treatments.

Notably, two indicators were common across all four pathways: the number of new cases presented at cancer multidisciplinary team meetings and the number of consultations with psychologists or psychiatrists.

These indicators collectively encompass all stages of the treatment process: diagnosis (10/49; 20.4%), treatment (36/49; 73.5%), staging (1/49; 2%), counseling (1/49; 2%), follow up (1/49; 2%), and therapeutic delays (2/49; 4.1%).

## 4. Discussion

The present study demonstrates the feasibility of developing a multidisciplinary set of QIs tailored to multiple cancer cohorts in the context of a global disruption of healthcare systems. We identified a total of forty-nine QIs across four cancer care pathways: twelve for breast cancer, eleven for hepatocellular carcinoma, eight for gynecological cancers, and eighteen for peritoneal carcinomatosis.

This approach to improving the quality and safety of care was guided by a steering committee and a dedicated team of experts. The establishment of a multidisciplinary team of clinicians allowed for the integration of diverse perspectives and strengthened collaboration among the participating hospitals. The selected indicators underwent professional consensus and align with the existing literature and best practice recommendations. The RAND/UCLA method provided a robust methodological framework for thoroughly evaluating each indicator. Through a comprehensive, structured, and evidence-based approach, we identified specific indicators relevant to the four cancers studied, covering the entire patient care pathway and facilitating coordination among various stakeholders.

Currently, these QIs are being assessed for their feasibility of implementation and their ability to reliably measure relevant outcomes.

Our study revealed a high proportion of process indicators (92%) compared to historical QI sets in oncology [15,21,22]. This finding can be attributed to our focus on indicators specifically designed to assess the effects of global healthcare disruptions, such as those experienced during the COVID-19 pandemic. Foundational indicators may have been considered less likely to be affected by such disruptions, as they primarily evaluate organizational dimensions, which fell outside the scope of this study. Quality impact indicators were selected less frequently, likely because many can only be assessed after a significant post-treatment interval, making them less effective for real-time monitoring of care quality.

Process indicators offer several advantages in this context. They can be readily extracted from patient records or other data sources (such as cohorts and registries), and some could potentially be derived from medico-administrative data [23]. These indicators are particularly useful for evaluating changes in healthcare practices [24] and identifying deficiencies in patient care over time. Importantly, only quality indicators supported by scientific evidence on process and outcome evaluation were selected. Additionally, process indicators are typically easier for healthcare providers to interpret, offering actionable insights that facilitate the replication of corrective interventions and the generation of generalizable knowledge for implementing complex healthcare improvements [25]. Beyond these general advantages, the indicators developed in this study were designed with the specific challenges of healthcare crises in mind. They focus on critical steps in the care pathway that are highly vulnerable to disruption, such as diagnostic and treatment delays. Their selection was based on feasibility for rapid data collection and real-time use, making them particularly relevant for monitoring care quality under emergency conditions [17,18]. The inclusion of a crisis-specific criterion in the RAND/UCLA validation process further ensures their contextual appropriateness, while their methodological simplicity supports future replicability and use in preparedness strategies.

The integration of both process and outcome indicators enables a comprehensive assessment of care relevance and coordination, thereby illustrating the tangible impact of the COVID-19 crisis and potential future crises. It is crucial to recognize that all indicator classifications are interconnected; thus, indicators should not be analyzed in isolation but rather within a holistic framework that considers the entire patient care pathway.

At this stage, the selected QIs have yet to be collected for the COVID-19 pandemic year and preceding years. Consequently, their reliability and feasibility for data collection remain to be evaluated. A previous study proposed breast cancer-specific QIs that were automated using the French real-life medico-administrative cancer database to develop a standardized set for breast cancer care [23]. In that study, 10 indicators were selected compared to 12 in our study, despite both being derived from similar international research [21]. Notably, only four QIs were common to both studies, highlighting the need to tailor indicator sets to their intended use and feasibility of data collection, as a standardized set may not be universally applicable across different healthcare settings.

A significant limitation of our study was the absence of patient partners in the expert panels due to the health context at the time of this study’s initiation. Including patients in this type of research is strongly recommended [25,26], as their perspectives could have fostered a more patient-centered approach. Their participation would have allowed for a better consideration of key aspects of patients’ lived experiences, such as their journey through the care pathway and the impact on their quality of life. Their absence may, therefore, have influenced the weighting or selection of certain indicators closely linked to patients’ subjective perceptions, particularly those related to treatment delays or access to supportive care services [27]. As such, the selected QIs will require validation by patient panels in future studies.

Additionally, it is important to note that all experts involved were affiliated with French institutions, and the applicability of the selected QIs may vary in other national contexts.

While this study was conducted within the French healthcare system, adapting and validating the selected indicators in non-French-speaking or resource-limited contexts represents an important area for future work. Most of the process indicators focus on core steps of the oncology care pathway, such as diagnosis, treatment, and follow up, which are broadly applicable across healthcare systems. However, contextual adaptation will be necessary, including cultural and organizational validation, assessment of data collection feasibility, and alignment with local clinical relevance. A two-phase approach could be envisioned: initial adaptation through consultation with local experts and patient representatives followed by pilot testing to evaluate feasibility, reproducibility, and sensitivity to change in diverse settings.

The pandemic has underscored the critical importance of preparedness in the healthcare sector to ensure optimal patient care and equitable access to medical services. Developing indicators specifically designed to monitor care quality during periods of healthcare disruption is a key component of crisis preparedness. These indicators must be validated, implemented, and embraced by the medical community to be effectively utilized in future crises.

The next phase of this project will focus on assessing the reliability and reproducibility of the validated indicators, as well as evaluating the feasibility of real-time data collection. Conducting this study across four regional hospitals will provide a representative overview of the pandemic’s impact. Ultimately, the goal is to expand this research into an international study, introducing standardized indicators while considering the specific characteristics and cultural contexts of each country.

## 5. Conclusions

This study demonstrates the feasibility of developing crisis-responsive QIs to monitor cancer care during health system disruptions. Future work will focus on their real-time implementation, validation in international settings, and integration into healthcare policies to enhance crisis preparedness.

## Figures and Tables

**Table 1:** Expert panel compositions.

	Breast Cancer	Hepatocellular Carcinomatosis	Gynecologic Cancer	Peritoneal Carcinomatosis
Bibliographic panel composition	Medical oncologist, *n* = 1 Surgeons, *n* = 2	Gastroenterologist, *n* = 3	Medical oncologist, *n* = 1 Surgeons, *n* = 2	Surgeons, *n* = 2
	Years of experience <10, *n* = 3 10–20, *n* = 0 >20, *n* = 0	Years of experience <10, *n* = 2 10–20, *n* = 1 >20, *n* = 0	Years of experience <10, *n* = 2 10–20, *n* = 1 >20, *n* = 0	Years of experience <10, *n* = 1 10–20, *n* = 0 >20, *n* = 1
Expert panel composition	Medical oncologist, *n* = 2 Surgeons, *n* = 3 Radiation therapist, *n* = 1	Medical oncologist, *n* = 1 Gastroenterologist, *n* = 6	Medical oncologist, *n* = 1 Surgeons, *n* = 3 Radiation therapist, *n* = 2	Surgeons, *n* = 5
	Years of experience <10, *n* = 0 10–20, *n* = 3 >20, *n* = 3	Years of experience <10, *n* = 0 10–20, *n* = 1 >20, *n* = 6	Years of experience <10, *n* = 1 10–20, *n* = 2 >20, *n* = 3	Years of experience <10, *n* = 1 10–20, *n* = 2 >20, *n* = 2

## Data Availability

Data are contained within the article (See Appendix A).

## Abbreviations

The following abbreviations are used in this manuscript:

QIs	Quality indicators

## Appendix A

Indicators Selected for the Four Cohort Cancers

**Table A1:** Specific quality of care indicators for breast cancer.

Indicator Title	Type of Indicator	Numerator	Denominator	Inclusion Criteria	Exclusion Criteria	Data Sources	Bibliographic References
Stage at diagnosis	Care process Staging	Number of women at each stage by UICC 8th edition	Number of women diagnosed with invasive non-metastatic breast cancer	Age > 18 Invasive non-metastatic carcinoma of the breast	Cancer at another location during the year Neoadjuvant chemotherapy or hormone therapy	Electronic Health Record (EHR)	[28,29]
Proportion of women with MRI in invasive lobular carcinoma	Care process Diagnosis	Number of patients with non-metastatic invasive lobular carcinoma who had an MRI with treatment	Number of patients treated for non-metastatic invasive lobular carcinoma	Age > 18 Diagnosis of invasive lobular carcinoma	Metastatic cancer	EHR SNDS (National Health Data System)	[30]
Number of women diagnosed with invasive non-metastatic breast cancer/week	Care process Diagnosis	Number of women diagnosed with invasive non-metastatic breast cancer/week	Number of women diagnosed with breast cancer/week	Age > 18 Invasive non-metastatic breast carcinoma		EHR	Added by the bibliographic panel.
Proportion of women who had their first treatment (surgery, chemotherapy, or hormone therapy) within 6 weeks or less of the date of the mammogram preceding treatment	Care process Therapeutic delay	Number of women who had their first treatment within 6 weeks or less of the date of the mammogram	Number of women having had a mammogram followed by treatment	Age > 18 Ductal carcinoma in situ or invasive non-metastatic invasive carcinoma of the breast Have had a mammogram Have had a biopsy Have received at least one treatment	History of contralateral breast cancer Other cancers diagnosed in the same year Chaining problem between different AMDB stays	EHR SNDS	[21,31]
Proportion of women with ductal carcinoma in situ or invasive non-metastatic breast cancer whose time between surgery and first additional treatment (chemotherapy or radiotherapy) is within the expected timeframe	Care process Therapeutic delay	Time between surgery and radiotherapy < 12 weeks of radiotherapy Time between surgery and chemotherapy < 6 weeks if chemotherapy	Invasive breast carcinoma (CIM-10): C50; C500; C506; C508; C509 + Chirurgie (CCAM): QEFA001; QEFA003; QEFA004; QEFA005; QEFA007; QEFA008; QEFA010; QEFA012; QEFA013; QEFA017; QEFA020. QEFA015	Age > 18 Invasive non-metastatic breast carcinoma operated on and having had additional treatment	History of contralateral breast cancer Cancer at another location during the year	SNDS	[30,32,33,34,35]
Proportion of neoadjuvant chemotherapy	Care process Treatment	Number of women receiving intravenous chemotherapy before surgery for invasive breast cancer	Number of women diagnosed with invasive non-metastatic breast cancer	Age > 18 Invasive non-metastatic breast carcinoma	History of breast cancer Cancer at another location during the year	SNDS	[32]
Time between biopsy and first surgery (excluding neoadjuvant chemotherapy)	Care process Therapeutic delay	Number of women who had surgery within 4 weeks or less of the date of the pathological report	Number of women who had a mammogram followed by a diagnosis of invasive non-metastatic breast cancer treated by primary surgery (no neoadjuvant chemotherapy or hormone therapy) during the given period	Age > 18 Invasive non-metastatic breast carcinoma	Neoadjuvant chemotherapy or hormone therapy Cancer at another location during the year	SNDS	[21,30]
Proportion of women for whom the time between the end of adjuvant chemotherapy and the start of radiotherapy was less than 6 weeks	Care process Therapeutic delay	Number of women with invasive non-metastatic breast carcinoma who had radiotherapy within 6 weeks of completing adjuvant chemotherapy	Number of women with invasive non-metastatic breast carcinoma who have had chemotherapy followed by radiotherapy	Age > 18 Invasive non-metastatic breast carcinoma Have had adjuvant chemotherapy Have had radiotherapy after adjuvant chemotherapy	History of contralateral breast cancer Cancer at another location during the year Surgical revision (mastectomy or axillary dissection) between chemotherapy and radiotherapy	SNDS	[30]
Proportion of women with a delay between the initial biopsy and the first surgery of less than 3 months	Care process Therapeutic delay	Number of women a delay between the initial biopsy with a diagnosis of cancer and surgery below 3 months	Number of women with invasive non-metastatic breast carcinoma treated with upfront surgery	Age > 18 Invasive non-metastatic breast carcinoma with upfront surgery	Cancer at another location during the year	EHR SNDS	[21,31]
Proportion of women who received radiotherapy after breast-conserving surgery for ductal carcinoma in situ or invasive non-metastatic breast cancer	Care process Treatment	Number of women with ductal carcinoma in situ or invasive non-metastatic breast cancer who had radiotherapy after conservative surgery	Number of women with ductal carcinoma in situ or invasive non-metastatic breast cancer who have undergone conservative surgery	Age > 18 Ductal carcinoma in situ or invasive non-metastatic breast cancer	History of contralateral breast cancer Cancer at another location during the year	SNDS	[30]
Number of consultations with a psychologist or psychiatrist	Care process Treatment	Number of consultations with a psychologist or psychiatrist (SF-12 mental, HAD, EORTC)	Number of women with ductal carcinoma in situ or invasive non-metastatic breast cancer	Age > 18, justifying psychological, psychiatric, or psychiatric care	_	EHR	Added by the bibliographic panel
Number of new files presented to specialist MTDMs	Care process Diagnosis	Number of new files presented to specialist MTDMs	Number of women diagnosed with breast cancer	All new patients presented to specialist MTDMs for breast cancer	_	EHR	Added by the bibliographic panel

**Table A2:** Specific quality of care indicators for hepatocellular carcinoma (HCC).

Indicator Title	Type of Indicator	Numerator	Denominator	Inclusion Criteria	Exclusion Criteria	Data Sources	Bibliographic References
Proportion of patients diagnosed with hepatocellular carcinoma (HCC) who received curative treatment	Care process Treatment	Number of patients with HCC receiving curative treatment (resection/local ablation/liver transplantation, LT)	Total number of patients with HCC	All new HCC + resection/local ablation/LT within 1 year	_	SNDS (MID) Health data warehouse (HDW)	[36,37,38,39]
Proportion of patients on the HCC transplant waiting list eligible for waiting treatment and treated during the waiting phase	Care process Treatment	Number of patients on the transplant waiting list for active HCC with treatment on hold	Number of patients on the transplant waiting list with active HCC and preserved liver function	Patient on the transplant waiting list with active HCC	Registration with a “non-treatable HCC” component	ABM, excluding an untreatable HCC component	[36,37,38,39]
Proportion of patients receiving post-treatment monitoring after resection or TPC	Care process Treatment	Number of patients with cross-sectional abdominal imaging/3 months within 2 years of resection/local ablation	Number of patients treated by resection/local ablation/ transplant	Patient treated by resection/local ablation/ transplant	_	EHR	[36,40]
Perioperative mortality (90 days) after liver resection in cirrhotic patients	Quality impact	Number of deaths within 90 days of HCC resection	Number of resections for HCC	All new HCC + surgical resection	_	SNDS	[39]
Perioperative mortality (90 days) after liver transplantation for HCC	Quality impact	Number of deaths within 90 days of liver transplantation for HCC	Number of transplants for HCC	Liver transplant for HCC	_	SNDS	[39]
Number of new HCC files presented to specialist liver MDTMs	Care process Diagnosis	Number of new HCC files presented to specialist liver MDTMs	Number of patients diagnosed with HCC	All new HCC presented to the liver specialist MDTMs	_	EHR	[41]
Time between first diagnostic imaging and MDTM presentation	Care process Diagnosis	Time between the first imaging describing a liver nodule and the date of the first MDTM presentation	Number of patients with a first imaging exam describing a liver nodule who were subsequently presented at an MDTM during the given period	All new HCC presented to the liver specialist MDTMs	_	EHR	[42]
Time between first MDTMs and first treatment	Care process Therapeutic delay	Time between the first presentation at MDTMs and the first treatment	Number of patients who were presented at an MDTM and subsequently received a first treatment during the given period	All new HCC presented to the liver specialist MDTMs	_	EHR	[42]
Percentage of patients with histological evidence of HCC	Care process Diagnosis	Percentage of patients with histological confirmation of HCC	Total number of patients diagnosed with HCC	All new HCC presented to the liver specialist MDTMs	_	DPI	[42]
Proportion of patients on the list for HCC transplants	Care process Treatment	Number of patients transplanted for HCC	Number of patients on the list for HCC	Patient on the list for HCC	MELD > 20	ABM	[36,37,38,39]
Number of consultations with a psychologist or psychiatrist	Care process Treatment	Number of consultations with a psychologist or psychiatrist (SF-12 mental, HAD, EORTC)	Number of patients treated for HCC	Age > 18, justifying psychological, psychiatric, or psychiatric care	_	EHR	Added by the bibliographic panel

**Table A3:** Specific quality of care indicators for gynecological cancer (excluding ovarian cancer).

Indicator Title	Type of Indicator	Numerator	Demoninator	Inclusion Criteria	Exclusion Criteria	Data Sources	Bibliographic References
Time between the date of surgery and the date of the first radiotherapy session	Care process Therapeutic delay	Time between surgery and adjuvant radiotherapy	Number of patients with gynecological cancer who underwent surgery and received adjuvant radiotherapy during the given period	Woman with gynecological cancer (excluding ovarian cancer)	_	SNDS	[43]
Annual percentage of women treated with radiotherapy or radiochemotherapy as first-line treatment for cervical cancer	Care process Treatment	Annual number of women with cervical cancer receiving radiotherapy or radiochemotherapy	Annual number of women diagnosed with cervical cancer	Woman with with cervical cancer in the first-line treatment	_	SNDS	[44]
Time between first consultation and date of biopsy	Care process Diagnosis	Time between first consultation and date of biopsy	Number of women diagnosed with gynecological cancer during the given period	Age > 18, requiring diagnostic biopsy	Biopsy available at the first consultation	EHR	Added by the expert panel.
Time between diagnosis and surgery for cervical cancer	Care process Therapeutic delay	Time between diagnosis and surgery	Number of women diagnosed with cervical cancer and treated with upfront surgery during the given period	Hysterectomy or trachelectomy for stage Ia-IIa cervical cancer	Pre-cancerous cells Surgery other than hysterectomy or trachelectomy	EHR SNDS	[45,46]
Percentage of patients for whom surgery is indicated who have received neoadjuvant treatment	Care process Treatment	Number of patients receiving neoadjuvant treatment (radiotherapy, chemotherapy, hormone therapy)	Number of patients with a surgical indication during the given period	Woman with gynecological cancer (excluding ovarian cancer)	Patient with no indication for surgery	EHR	[47]
Time between the first symptom reported by the patient and the first consultation	Care process Diagnosis	Time between first symptom and first consultation in gynecology	Number of women diagnosed with gynecologic cancer and with at least one reported symptom at the time of diagnosis during the given period	Woman with gynecological cancer (excluding ovarian cancer)	_	EHR	Added by the expert panel.
Number of new files presented to specialist MDTMs	Care process Diagnosis	Number of new files presented to specialist MDTMs	Number of newly diagnosed cancer patients	All new women presented to specialist MDTMs	_	EHR	[48]
Number of consultations with a psychologist or psychiatrist	Care process Treatment	Number of consultations with a psychologist or psychiatrist (SF-12 mental, HAD, EORTC)	Number of patients treated for cancer	Age > 18, justifying psychological, psychiatric, or psychiatric care	_	EHR	[48]

**Table A4:** Specific quality of care indicators for peritoneal carcinomatosis.

Indicator Title	Type of Indicator	Numerator	Denominator	Inclusion Criteria	Exclusion Criteria	Data Sources	Bibliographic References
Time between consultation for curative indication and CRS +/− HIPEC	Care process Therapeutic delay	Time between consultation for curative indication and CRS ± HIPEC	Number of patients who had a consultation for curative intent and underwent CRS ± HIPEC during the given period	Age > 18 Resectable peritoneal carcinosis of digestive or gynecological origin	Age < 18 Unresectable peritoneal carcinosis of digestive or gynecological origin	EHR SNDS	Added by the bibliographic panel.
Time between MDTM decision and CRS +/− HIPEC	Care process Therapeutic delay	Time between MDTM decision and CRS ± HIPEC	Number of patients for whom CRS ± HIPEC was decided at an MDTM and performed during the given period	Age > 18 Resectable peritoneal carcinosis of digestive or gynecological origin	Age < 18 Unresectable peritoneal carcinosis of digestive or gynecological origin	EHR SNDS	Added by the bibliographic panel.
Time without treatment (chemotherapy or CRS +/− HIPEC)	Care process Therapeutic delay	Time without treatment (chemotherapy or CRS +/− HIPEC)	Number of patients who had a documented treatment interruption or treatment delay during the given period	Age > 18 Resectable peritoneal carcinosis of digestive or gynecological origin	Age < 18 Unresectable peritoneal carcinosis of digestive or gynecological origin	EHR SNDS	Added by the bibliographic panel.
Proportion of interventions (CRS +/− HIPEC) postponed	Care process Treatment	Number of patients operated on	Total number of patients planned for CRS +/− HIPEC	Age > 18 Resectable peritoneal carcinosis of digestive or gynecological origin with CRS +/− HIPEC postponed	Age < 18 Unresectable peritoneal carcinosis of digestive or gynecological origin	EHR SNDS	Added by the bibliographic panel.
Proportion of conversions to non-resectability	Care process Treatment	Number of exploratory laparotomies without CRS +/− HIPEC	Total number of patients planned for CRS +/− HIPEC	Age > 18 Peritoneal carcinosis of digestive or gynecological origin, which has become unresectable due to waiting times	Age < 18 Resectable peritoneal carcinosis of digestive or gynecological origin	EHR	[49,50]
Proportion of patients progressing after deferral or cancellation (morphological assessment/markers)	Quality impact	Number of patients with clinical, biological, or morphological progression	Total number of patients planned for CRS +/− HIPEC	Age > 18 Peritoneal carcinosis of digestive or gynecological origin with clinical, biological, or morphological progression due to waiting time	Age < 18	EHR	[51,52]
Total duration of chemotherapy (weeks) or number of cycles of chemotherapy	Care process Treatment	Number of chemotherapy cycles administered per patient	Number of patients who received chemotherapy during the given period	Age > 18 Resectable peritoneal carcinosis of digestive or gynecological origin	Age < 18 Unresectable peritoneal carcinosis of digestive or gynecological origin	SNDS	[53]
Rate of additional cycles of chemotherapy compared with the initial number	Care process Treatment	Number of additional chemotherapies	Number of chemotherapy treatments initially planned	Age > 18 Resectable peritoneal carcinosis of digestive or gynecological origin	Age < 18 Unresectable peritoneal carcinosis of digestive or gynecological origin	EHR SNDS	Added by the bibliographic panel.
Proportion of patients cancelled on the same day	Care process Treatment	Number of patients cancelled on the same day	Number of chemotherapy treatments initially planned	Age > 18 Resectable peritoneal carcinosis of digestive or gynecological origin	Age < 18 Unresectable peritoneal carcinosis of digestive or gynecological origin	EHR	[54,55]
Proportion of patients with shortened prehabilitation (<3 weeks)	Care process Treatment	Number of patients planned for CRS +/− HIPEC with prehabilitation < 3 weeks	Number of chemotherapy treatments initially planned	Age > 18 Resectable peritoneal carcinosis of digestive or gynecological origin	Age < 18 Unresectable peritoneal carcinosis of digestive or gynecological origin	EHR	[50]
Morbidity–mortality rate within 30 days of surgery after CRS +/− HIPEC	Quality impact	Number of patients who experienced severe post-operative complications or died within 30 days after CRS ± HIPEC	Number of patients who underwent CRS ± HIPEC during the given period	Age > 18 Resectable peritoneal carcinosis of digestive or gynecological origin	Age < 18 Unresectable peritoneal carcinosis of digestive or gynecological origin	SNDS	[56]
Time between consultation for indication of PIPAC and first PIPAC	Care process Therapeutic delay	Time between consultation for indication of PIPAC and first PIPAC	Number of patients for whom PIPAC was indicated and performed during the given period	Age > 18 Unresectable peritoneal carcinosis of digestive or gynecological origin	Age < 18 Resectable peritoneal carcinosis of digestive or gynecological origin	EHR SNDS	Added by the bibliographic panel.
Time between MDTM decision (indication given) and first PIPAC	Care process Therapeutic delay	Time between MDTM decision (indication given) and first PIPAC	Number of patients for whom a PIPAC was indicated in an MDTM and performed during the given period	Age > 18 Unresectable peritoneal carcinosis of digestive or gynecological origin	Age < 18 Resectable peritoneal carcinosis of digestive or gynecological origin	EHR SNDS	Added by the bibliographic panel.
Time without active treatment (chemotherapy or surgery) during the therapeutic pathway	Care process Therapeutic delay	Time without active treatment (chemotherapy or surgery) during the therapeutic pathway	Number of patients treated during the given period	Age > 18 Unresectable peritoneal carcinosis of digestive or gynecological origin	Age < 18 Resectable peritoneal carcinosis of digestive or gynecological origin	EHR	[53]
Proportion of interventions (PIPACs) reported	Care process Treatment	Actual number of patients operated on	Total number of patients included in the PIPAC pathway	Age > 18 Unresectable peritoneal carcinosis of digestive or gynecological origin	Age < 18 Resectable peritoneal carcinosis of digestive or gynecological origin	EHR	Added by the bibliographic panel.
Proportion of patients treated with PIPAC alone	Care process Treatment	Number of patients treated by PIPAC alone	Total number of patients included in the PIPAC pathway	Age > 18 Unresectable peritoneal carcinosis of digestive or gynecological origin	Age < 18 Resectable peritoneal carcinosis of digestive or gynecological origin	SNDS	Added by the bibliographic panel.
Number of new files presented to specialist MDTMs	Care process Diagnosis	Number of new files presented to specialist MDTMs	Number of new patients diagnosed	All new patients presented to specialist MDTMs	_	EHR	[48]
Number of consultations with a psychologist or psychiatrist	Care process Treatment	Number of consultations with a psychologist or psychiatrist (SF-12 mental, HAD, EORTC)	Number of patients treated for cancer	Age > 18, justifying psychological, psychiatric, or psychiatric care	_	EHR	[48,50]
